# Energy-Balanced Multisensory Scheduling for Target Tracking in Wireless Sensor Networks

**DOI:** 10.3390/s18103585

**Published:** 2018-10-22

**Authors:** Juan Feng, Hongwei Zhao

**Affiliations:** 1 School of Aerospace Science and Technology, Xidian University, Xi’an 710071, China; 2 School of Electronic Information, Northwestern Polytechnical University, Xi’an 710072, China

**Keywords:** target tracking, energy balance, multisensory scheduling, WSNs

## Abstract

One important way to extend the lifetime of wireless sensor networks (WSNs) is to manage the sleep scheduling of sensor nodes after they are deployed. Most of the existing works on node scheduling mainly concentrate on nodes which have only one sensor, and they regard a node and its sensor modules as a whole to manage sleep scheduling. Few works involve the sensed modules scheduling of the sensor nodes, which have multiple sensors. However, some of the sensed modules (such as video sensor) consume a lot of energy. Therefore, they have less energy efficiency for multisensory networks. In this paper, we propose a distributed and energy-balanced multisensory scheduling strategy (EBMS), which considers the scheduling of both the communication modules and the sensed modules for each node in target tracking WSNs. In EBMS, the network is organized as clustering structures. Each cluster head adaptively assigns a sleep time to its cluster members according to the position of the members. Energy-balanced multisensory scheduling strategy also proposes an energy balanced parameter to balance the energy consumption of each node in the network. In addition, multi-hop coordination scheme is proposed to find the optimal cooperation among clusters to maximize the energy conservation. Experimental results show that EBMS outperformed the state-of-the-art approaches.

## 1. Introduction

Wireless sensor networks (WSNs) consist of a large amount of small, low-cost, and wirelessly connected sensor nodes deployed in an unattended natural environment. Target tracking is a classical application in WSNs. Recently, because of sensor technological advances, sensors have become inexpensive and diversified. Therefore, the use of sensor node with multiple sensors for target tracking has become common. Because the battery of each sensor node is energy limited, it is difficult to replace the battery manually after the network has been deployed. Thus, optimization and balance of the energy cost for multiple sensors have become increasingly important [1,2].

The scheduling management for each module of sensor nodes is one common approach to conserving energy after the network is deployed [3]. When all the modules of a sensor node are in sleep mode, the node cannot sense and communicate so that it consumes extremely low power. Consequently, the main idea of sensor scheduling management is to keep a minimal number of sensor nodes or their modules active, in order to provide a necessary coverage of the sensing field, and to put the other nodes or their modules in sleep mode to save energy. Some existing research assign each sensor node a sleep time, and periodically put nodes to sleep, such as References [4,5]. This kind of sleeping scheme is easy to implement but it cannot adapt to the dynamic features of target tracking applications. If a target suddenly changes its direction, it does not work well. Instead, some coordinate approaches suit better by dynamically adjusting the sleep schedule of each node using information from neighboring nodes such as in References [6,7,8]. These coordinated approaches have a superior real-time performance and estimation accuracy. However, the frequent communication among neighboring nodes introduce a large overhead on traffic load, and unsynchronized sleeping of the nodes increases the end-to-end delay of multi-hop data transmission. Furthermore, these schemes consume more energy, and the energy distribution of the whole network is not balanced.

In addition, the existing research on node management and scheduling for target tracking mainly concentrate on the nodes which just have one sensor, and few studies pay attention to the sensed modules scheduling for multisensory nodes. The existing research considers the radio of each node as the module which consumes the most energy. Nevertheless, with the development of sensing technology, sensor nodes in WSNs are usually equipped with multiple sensors nowadays, and the sensors can bring different sensing signals, such as temperature, infrared, distance, video, and so on. Utilizing the sensing observations from multiple sensors to coordinate the target tracking tasks is a very important way in the future, and it can achieve better tracking performance than using the sensing observation from a single sensor. Therefore, the scheduling management of sensed modules must be taken into consideration. Management sensed modules for each node affects not only the energy consumption of the node, but also the storage and transmission of the sensed data. For example, video sensors consume more energy and bandwidth in data transmitting, which is also an important aspect of the whole network performance. 

With the above motivations, in this paper, we propose a novel distributed and energy-balanced multisensory scheduling strategy, called EBMS, for target tracking WSNs. The major objective of EBMS is to manage the sleep or active status of the multiple sensed and communication modules for each sensor node so as to track a target using less energy. In EBMS, the network is organized as clustering structures. Each cluster head (CH) adaptively assigns a sleep time to its cluster members (CMs) according to the distance between the member and the cluster border. Also, CH estimates transferred factors for its CMs based on its gathered information from its members and neighbor CHs, and then each CM decides whether its modules can transfer into active in the next time step or not. In addition, this paper optimizes the CHs cooperation for maximum energy efficiency. The contributions in this work are detailed as follows:

- We propose a novel and distributed multiple sensors scheduling approach. The other existing works did not involve the sensed modules scheduling for one sensor node with multiple sensors, and they regarded the nodes and sensed modules as a whole to manage the sleep scheduling. This work considers not only the scheduling management of communication modules, but also the sleep scheduling of each sensed module in one node based on their power consumption and sensing performance. Thus, both the nodes and their sensed modules can be efficiently scheduled.
- This paper proposes an adaptive sleep time allocation scheme to maximize the sleep time of each CM, and each CH can adaptively arrange the sleep time of its members according to the distance between the member and the cluster border. Moreover, the collaboration is established among CHs in our coordinated scheme.
- This paper proposes an energy balanced parameter to balance the energy consumption of each node and proposes a target state uncertainty factor to guarantee the tracking performance. Furthermore, each node receives the transferred factors from its CH to estimate the probability of its sensed modules entering active mode in the next time step. Consequently, EBMS can achieve energy consumption balance for each sensor node.
- A multi-hop coordination scheme among CHs is proposed to find the optimal cooperation among CHs and maximize the energy conservation. Moreover, an optimal sleep scheduling for the nodes and their modules is obtained by the multi-hop cluster coordination. As a result, the nodes and sensed modules can be woken up in time to avoid missing a target when the target moves dynamically and randomly.

The rest of this paper is organized as follows. A brief review of related works is introduced in Section 2. The network and sensor node models are presented in Section 3. Then Section 4 presents the proposed approach in detail. The simulation results are given in Section 5, and the conclusions are made in Section 6.

## 2. Related Work

To minimize energy consumption and extend network lifetime, a common technique is to put some sensors in sleep mode and put others in active mode for the sensing and communication tasks [3]. A simple node scheduling approach in view of data correlation (SSA-DC) is proposed in Reference [4]. The authors divide the networks into clusters using an adaptive dual-metric K-means (DK-means) method. In each cluster, the node works as a group leader in turns and sends data to sink. In Reference [5], the authors propose a service-oriented node scheduling approach to provide multiple sensing services while maximizing the network lifetime, and they build a data correlation model for different services by using Markov random field. However, these schemes are not flexible since all nodes periodically wake up for a fixed time. To be more flexible, an energy efficient node scheduling mechanism has been proposed in Reference [9] that uses the concept of lightweight processors. The idea is to cut down the processor into two parts: the main processor and the lightweight processor. The main processor contains the major part of the processor, whereas the lightweight processor contains a small portion, which comes into operation only when a chance of missing a deadline is hit.

In Reference [10], the authors propose a random independent scheduling (RIS) to extend network lifetime while achieving asymptotic K-coverage. Random independent scheduling assumes that time is divided into cycles based on a time synchronization method. At the beginning of a cycle, each node independently decides whether to become active with probability *p* or go to sleep with probability 1 − *p*. In Reference [11], an optimized back-off sleep protocol (OBSP) is proposed, which allows nodes to independently determine their scheduling mechanism based on a battery discharge curve of active nodes. Due to a battery discharge rate, OBSP avoids unnecessary and random frequent wake-ups at lower residual energy in the sleeping nodes. In Reference [12], the authors propose a node management method by a sleep control game, in which each node selects the best wake-up strategy according to the payoff function that may minimize the energy consumption and maintain the latency performance. However, due to the limited sensed ability of a single node, in these independent scheduling methods the node cannot adjust its sleeping state according to the target information in time.

Then, a distributed saturation degree-based algorithm (DSDA) is proposed in Reference [13], which transformed the scheduling problem into a coloring problem by graph theory, and the node scheduling can be implemented locally by each node with information collected from its neighbors. The authors in Reference [6] propose an adaptive coordinated sleep scheduling scheme, where each node periodically broadcasts a message to its neighbors to indicate whether there is a target being detected. In this way, nodes are aware of the status of their neighbors. In Reference [7] the authors propose a sleep schedule with a service coverage guarantee by considering the redundancy degree on both the service level and the node level. Furthermore, they propose the sleep mechanism based on a sleep factor which integrates the redundancy degree, reliability, and energy. In Reference [8], the authors propose an algorithm to solve the problem of node hibernation for the heterogeneous sensing coverage areas. They use an appropriate and collaborative mechanism to conserve power in the distributed constraint optimization. Although a node can decide its sleep mode based on the information from its neighbors in these coordinated approaches, because the nodes in WSNs are dense deployment, the frequent broadcasts information to their neighbors will dramatically increase the traffic load and lead to congestions and high energy consumption. 

A prediction-based on sleep scheduling protocol is proposed in Reference [14], which predicts the position of a target by a particle filter. In Reference [15], the authors propose a probability-based prediction and sleep scheduling protocol (PPSS), in which a target prediction method based on both kinematics and probability is designed to precisely awaken the selected nodes. In Reference [16], the authors propose a distributed node-level energy management approach called prediction-based opportunistic sensing (POSE), in which the network self-adapt to target trajectories by enabling high-power consuming devices when they predict that a target is arriving in their coverage area, while enabling low-power consuming devices when the target is absent. 

In a densely deployed WSN, since only a few nodes need to keep active to have significant observations, to select and decide which sensors execute the sensing tasks are important. In Reference [17], the sensor selection scheme is transformed to the recovery of a sparse matrix. In Reference [18], a sparsity-aware sensor selection problem is formulated by minimizing the number of the selected sensors. In [19], the authors propose a node selection method by maximum entropy fuzzy clustering. They divided the problem of node selection into two levels, sensor-level tracking and global-level fusion. In Reference [20], the authors establish a linear system of equations to select sensor nodes, and the independent variables of the equation are the chosen sensors and the constraints include node number, the power or the sensing range of sensors and so on. In Reference [21], the problem of periodic sensor scheduling is addressed by seeking the optimal sparse estimator gain, where a one-to-one correspondence between active sensors and the non-zero columns of the estimator gain is established. For a more complete literature review on sensor management for target tracking, see Reference [21] and references therein. Moreover, in Reference [22] the authors propose a hierarchically energy management method that divides the network into hierarchical layers. Just one layer is in tracking state in one time instant and the others are in sleep state to save energy. However, since the central sink controls the type of hierarchical layer and sleep strategy in each hierarchical layer, the information of moving target needs to be transmitted to the sink in time to make a decision.

From the above analysis, we can see that few existing works considerate the scheduling management of the sensed modules for each sensor node in the target tracking WSNs, and most of the existing works regard the nodes and sensed modules as a whole to manage the sleep scheduling. Therefore, they have less energy efficiency for the multisensory networks.

## 3. The System Models

### 3.1. Network Model

We consider a WSN consisting of *n* sensor nodes which are deployed randomly and uniformly in a region of interest of size *a* × *b*. We assume that the target and all the sensors are in a two-dimensional deployed area. The position of node *N_i_* (1 ≤ *i* ≤ *n*) denoted by *X_i_*(*x_i_*,*y_i_*), is assumed to be known after deployment using GPS or any localization algorithm. As long as all the node locations are known in advance, our proposed approach can handle any node deployment pattern.

The sink has an infinite power supply as well as fixed location, and it gathers the sensing data from the whole sensor network. Each node is powered by the battery and energy constrained.

### 3.2. Sensing Model

We assume that each sensor node is equipped with multiple sensed modules *M_j_* (1 ≤ *j* ≤ *m*) and each sensor has a certain sensing range *R_s_*(*M_j_*). Denote *X_t_*(*t*) to be the target position at time step *t*, and the measurement *Z*(*M_j_*,*t*) of sensed module *M_j_* at time *t* can be expressed as following,

(1) $$Z(M_{j},t)=h(M_{j},t)+s(M_{j},t),j\in [1,m]\;$$

where *h*(*M_j_*,*t*) is the observation value of the sensed module *M_j_* of the node *N_i_* at the *t*-th time step, and *s*(*M_j_*,*t*) is the observation noise at the module *M_j_*. It is an independent and identically distributed Gaussian random variable with mean zero and covariance $\delta^{2}$. Then, the measurement *Z*(*N_i_*,*t*) of node *N_i_* can be express as,

(2) $$\begin{array}{ll} Z(N_{i},t) & =\{Z(M_{1},t),Z(M_{2},t),\ldots ,Z(M_{m},t)\}=H(M,t)+S(M,t)\\ & =\left[\begin{array}{l} h(M_{1},t)\\ h(M_{2},t)\\ \cdot \\ \cdot \\ \cdot \\ h(M_{m},t) \end{array}\right]+\left[\begin{array}{l} s(M_{1},t)\\ s(M_{2},t)\\ \cdot \\ \cdot \\ \cdot \\ s(M_{m},t) \end{array}\right],i\in [1,n] \end{array}$$

For convenience, the following definitions are made in this paper.

**Definition** **1.** *Neighbor Node Set (NN_i_). The neighbor node set of node N_i_ is denoted as NN_i_ = {N_l_ | d(N_i_,N_l_) ≤ R_m_, N_i_, ≠ N_l_}, where R_m_ is the minimum sensing radius of the sensed modules of N_i_ and d(N_i_,N_l_) is the Euclidean distance between node N_i_ and N_l_. In this paper, we assume N_l_* ∈ *NN_i_ when N_i_* ∈ *NN_l_, that is node N_i_ and N_l_ are neighbors each other.*

**Definition** **2.** *Tracking Candidates Set (TC). TC(t) is the set of the sensor nodes whose one or more sensed modules can detect the target at time t. If* $N_{i}\notin TC(t)$ *, node N_i_ cannot detect the target and has no sensor readings generated at time t.*

### 3.3. Sensor Node Model

**Figure 1:**
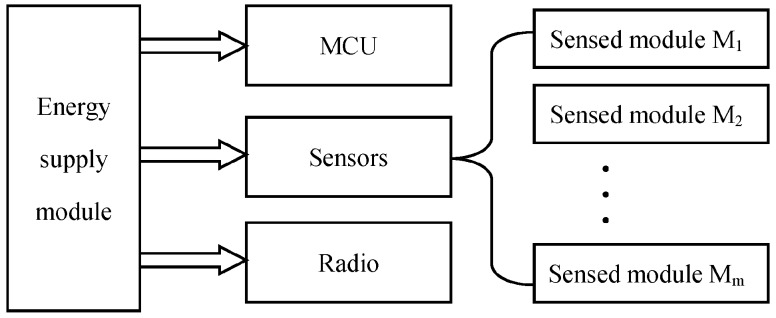
Structure of a sensor node.

A sensor node consists of communication, MCU (Microcontroller Unit), energy supply, and sensors, as Figure 1 shows, and the sensors include several multi-mode sensed modules. These modules have active and sleep states. As a result, each node has several power states according to the different combinations of each module state. A sensor node can be in one of the six states, sleep, idle, sensing, transition, receiving, and transmitting in which the latter five states are defined as active states. In idle state, sensor nodes do not sense, transmit or receive anything, but it consumes nearly as much energy as that of sensing, receiving or transmitting since the corresponding modules of the nodes are active, therefore the idle time of sensor nodes should be decreased as little as possible.

For communication module, it wakes up periodically from sleep state to receive data from the others. Table 1 shows some useful power states for the nodes, where the active sensing component means that there are one or more sensed module active and the sleep sensing component means that all the sensed module are sleep. The details of the states of the sensed modules are listed in Table 2.

In this paper, we consider the power management not only for the communication module but also the sensed module of the nodes, because the sensed module in active state can also consume more energy and at the same time produce large amounts of sensed data needed to be transmitted in the network. In most cases, not all the sensors have to keep active, thus it is necessary to make an appropriate power management for each node to decide which modules can be in sleep.

### 3.4. Energy Consumption Model

**Figure 2:**
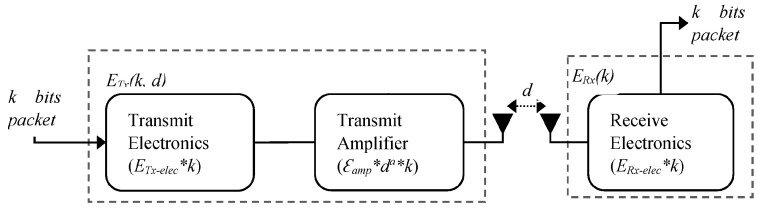
Energy cost model of the communication module.

An energy model is used in this work as Figure 2 shows. *E_Tx_*(*k*,*d*) and *E_Rx_*(*k*) are energy consumption of transmitting and receiving *k* bits data over a distance *d*.

(3) $$E_{Tx}(k,d)=(E_{Tx}{}_{-elec}+\varepsilon_{amp}*d^{\alpha })*k\;$$

(4) $$E_{Rx}(k)=E_{Rx-elec}*k\;$$

where *E_Tx_*_−*elec*_ and *E_Rx_*_−*elec*_ are the distance independent terms that are the overheads of transmitter and receiver electronics. *ε_amp_* [*Joule*/(*bit*·*m^α^*)] represents the energy needed to transmit one bit to achieve an acceptable signal to noise ratio over a distance *d*, and *α* is path loss exponent (2≤α≤5) which depends on the channel characteristics.

The sensed modules of sensor nodes have two statuses, active and sleep, and their energy consumption consist of three parts, working status, transition from active to sleep, and transition from sleep to active. The details can be shown as follows,

(5) $$E_{sens}=E_{a-s}+E_{s-a}+E_{acti}=G(e_{a-s}+e_{s-a})+P_{sens}T_{sens}\;$$

where *G* is the state switching times, *P_sens_* is the power of the active state. *T_sens_* is working time period. $E_{a-s}$ and $E_{s-a}$ are the energy consumption of node state transition from active to sleep and sleep to active respectively.

When a sensor node is in different states, it consumes different energy values because each module of the node has different states. When the node is in a specific state, each module of the node is also in a specific state. Therefore, we can estimate the energy consumption of each module based on the time period when the module is in the state and the power it consumes in the state. For example, for sensed modules, the power of each sensor in the active state is set in the simulation parameters, thus, the energy consumption of the sensor is calculated based on its power and active time period. Finally, the energy consumptions of all the modules in the node are added up to get the energy consumption of the node.

## 4. Energy-Balanced Multisensory Scheduling Algorithm

The existing research pay less attention to the power management for the sensor modules of each node. However, in EBMS, the power managements for both communication and sensor modules are considered, and the sensor management operations for a target tracking WSN are respectively carried out on CH and its members. If no target appears, all the sensor nodes need to keep a certain level of vigilance to prepare for detecting a target intrusion, and each node should turn off its modules as much as possible to save energy. Particularly, each node has to manage its multiple sensed modules to make a quick response to the target appearance with less energy consumption. Then the nodes around the target have to wake up to detect the movement of the target and report the sensed information to the sink. Dynamically arranging the sleep time for sensor nodes and the sensors are both necessary.

### 4.1. The Network Initialization and Clustering

After initialization, the network is organized as clusters, and in each cluster one node which has the most energy is selected as the CH. The cluster heads in this paper are the normal sensor nodes, and they do not have any external power sources. The nodes in a cluster take turns as CH. For achieving the desired detection accuracy, each CH calculates the sleeping time for sensor nodes and their modules and inform them via a wireless broadcasting channel. Since the nodes in one cluster have relatively short distances, each CM can directly connect to CH. However, among the CHs, we adopt the multi-hop fashion to transmit the data to the sink.

A residual energy threshold *th_ch_* for CH is set to control the frequency of the CH changed. In this work, *th_ch_* = 1/3 × E_ch_, and E_ch_ is the energy that the node has when the node is elected to be a CH. When the residual energy of the current CH is lower than the threshold, the CH sends a changing CH message to the other nodes in the cluster. After receiving the message of changing CH, the cluster member nodes in the cluster participate in the election of the next CH. Finally, the node with the most residual energy becomes the next CH in the cluster. When CH election fails, maybe because of the messages loss, the current CH will remain its role and broadcast the changing CH message periodically. For reliability purpose, when a CM fails to transmit data to its CH for several times (e.g., the CH dies suddenly), it will send a changing CH message to the CMs.

For all the clustering networks, there will be the overhead of the CH replacement. The overhead to update the information is caused by the current CH sending data to the next CH. Since the current CH only needs to send the information about its member and the cluster-related information to the next CH, the amount of data transmitting is relatively small and it will not bring too much overhead.

In clustering networks, the cluster structure will be re-organized after a certain time, which is determined by the application and the number of the exhausted nodes.

**Figure 3:**
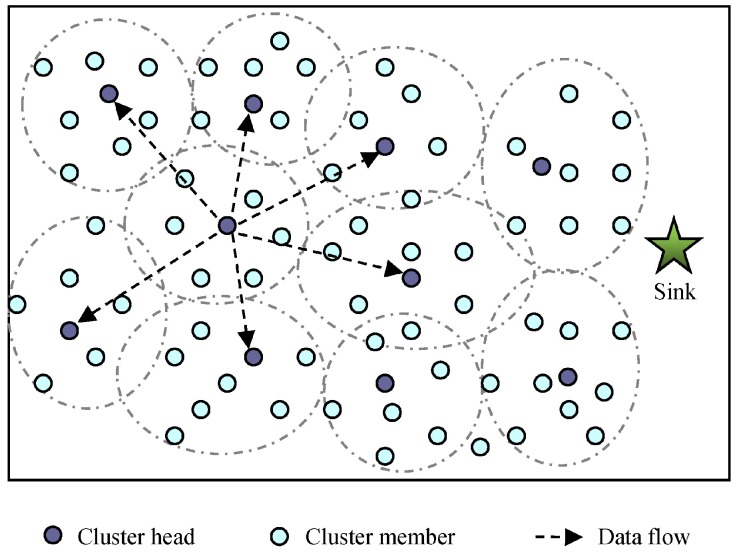
Illustration of sensor management in a clustering network.

In EBMS, the CH sends the observations of its own cluster to its neighbor CHs and receives the detected information from the neighbor CHs. The illustration of the sensor management in a clustering network is shown as Figure 3. Cluster members (CMs) remain their modules in sleep state most of the time and the modules change from sleep to active at specified time slots scheduled by its CH. In the active time slots, the communication module of the CM receives the assignment messages from its CH and the sensed module detects and checks if there are intruded targets. And then according to the received and sensed information, the node decides the probability of transferring into active for its each module in next time step. Therefore, the sensor management in EBMS consists of two steps, one is calculating the sleeping time for sensor nodes and their modules, and the other is estimating the probability of transferring into active state based on the gathered information for the next time step.

### 4.2. The Sleeping Time Calculation for Sensor Node and Its Modules

When the clusters are formed, each node sends its position information to its CH. After the CH collects all the position information of its members, it figures out the distance value between itself and each CM by the following equation,

(6) $$d(CH_{u},N_{i})=\sqrt{{\left(x_{u}-x_{i}\right)}^{2}+{\left(y_{u}-y_{i}\right)}^{2}}\;$$

where *d*(*CH_u_*,*N_i_*) is the distance between cluster head *CH_u_* and node *N_i_*, and (*x_u_*,*y_u_*) and (*x_i_*,*y_i_*) are the coordinates of *CH_u_* and node *N_i_* respectively. When the clusters are formed, each node sends its position information to its CH. Since each CH knows the location information of all its members and the distance value between itself and each CM, each CH can estimate the boundary range of the cluster and the distance between each CM and the cluster border $d(C_{b},N_{i})$.

As we know, if a target is going to enter the sensing area of a cluster, it has to first pass through the border of the cluster. In order to avoid missing a target, the sensor nodes near to the cluster border have to keep alert and have less sleep time than that of the nodes located in the cluster center. As a result, each CH can arrange the sleep time of its members according to the distance between the member and the cluster border. Concretely, the sleep interval of the CM *N_i_* can be calculated as the following equations,

(7) $$T_{s}(N_{i})=T_{s-\max}-\frac{d(C_{b},N_{i})}{\max\_d(CH_{u},C_{b})}(T_{s-\max}-T_{s-\min})-T_{tra}\;$$

(8) $$T_{s-\max}=\max\_d(CH_{u},C_{b})/v_{\max}\;T_{s-\min}=R_{s}(M_{e-\min})/v_{\max}\;$$

where *T_s_*_−*max*_ and *T_s_*_−*min*_ denotes the longest and the shortest sleep time in the cluster respectively, they are obtained from Equation (8). Moreover, max_*d*(*CH_u_*,*C_b_*) represents the maximum distance from the CH to the cluster border and *R_s_*(*M_e_*_−*min*_) represents the sensing range of the sensed module that has the least power cost among the sensed modules of the node. In addition, *v_max_* is the possible maximum target velocity that is decided according to the application, and *T_tra_* denotes the transition delay when the node changes from the sleep to active state. In addition, if the whole structure of the cluster is not changed and the position of the CMs is not changed, only the CH changes, then the sleep time of each CM remains unchanged. Therefore, the current CH sends the sleep information of its member to the next CH, which does not need to recalculate the sleep time of the members.

**Figure 4:**
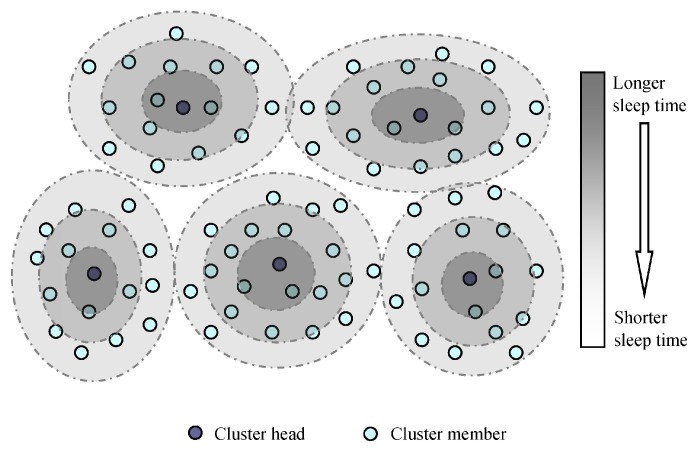
Illustration of the layers of the node sleep scheduling in clusters.

Using this way, the CMs near to the cluster center has longer sleep time while the CMs near to the cluster border has shorter sleep time, and then each CM is adaptively arranged different sleep time by its CH. Figure 4 illustrates the different sleep time of the CMs in each cluster. From the cluster center to border, the darker the color of the ring area is, the longer sleep time the node in the area has. As the color of the region changes from deep to shallow, the sleep time of the nodes in the corresponding area changes shorter. For balanced energy consumption, we can choose the nodes located in cluster center to execute the energy-intensive sensing or data transmitted task since these nodes can sleep more to save energy. We should notice that Figure 4 is just a sketch map that does not represent the actual sleep time of the CMs. Because of the random distribution of nodes, a cluster is not a regular shape, and the range of each cluster is not an accurate circle. Therefore, it is impossible to draw an accurate graph to represent the sleep time of the CMs. Figure 4 just indicates that the CMs at the border of the cluster sleep shorter, and the CMs at the center of the cluster sleep longer.

**Figure 5:**
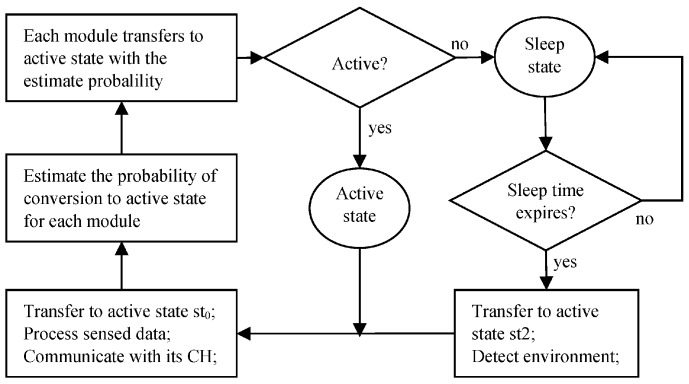
The procedure of sensor node and its modules scheduling.

After obtaining the sleep time value of each CM, the CH will inform its members about the sleep time and the assigned active time slots. For each member, it receives the sleep time assignment from its CH and gets into sleep sate. As the sleep time expires, the member node transfers to the active state st_2_, in which it just wakes up the sensor that has the least power cost among all the sensed modules in the node. Whereas the other sensed modules still keep in sleep state. And then the member node transfers to the active state st_0_ to process the sensed data and communicates with its CH. At the end of the current time step, the member node estimates the probability of conversion to active state for each module in the next time step according to the sensed and the received information. In the next time step, each module converts to active state with the estimated probability, and the modules which do not move into active continue sleeping for an assigned time period. And then the above process repeated as Figure 5 shows.

### 4.3. The Probability of Transferring into Active for Sensor Node and Its Modules

For each node in practice, the detection of a target is not always successful as long as the target is within the detection region of a sensor because of some reasons like time latency. Hence, we assume that the detection probability of the sensed module of a node is given as follow:

(9) $$P(M_{j})=\left\{ \begin{array}{ll} \lambda_{j} & X_{t}(t)\in R_{s}(M_{j})\\ 0 & X_{t}(t)\notin R_{s}(M_{j}) \end{array}\right.\;$$

where $0\leq \lambda_{j}\leq 1$ is a probability that sensed module *M_j_* can detect a target successfully if the target is within its detection region, *X_t_*(*t*) is the target’s position at *t* time step. If one of the sensed modules of node *N_i_* is in active state and detects a target, the detected result of *N_i_* will be set to 1. Otherwise if all the sensed modules of *N_i_* detect no target, the detected result will be set to 0, which shows in Equation (10).

(10) $$\left\{ \begin{array}{ll} dr(N_{i},t)=dr(M_{j},t)=1 & \;a\;\mathrm{target}\;\mathrm{detected}\\ dr(N_{i},t)=\sum_{j=1}^{m}dr(M_{j},t)=0 & \;\mathrm{no}\;\mathrm{target}\;\mathrm{detected} \end{array}\right.\;$$

where $dr(M_{j},t)$ denotes the detected results of *M_j_*, $dr(N_{i},t)$ is the detected results of node *N_i_* at *t* time step. If *M_j_* detects a target $dr(M_{j},t)=1$, or else $dr(M_{j},t)=0$.

After each node obtains its own sensed data in the current time step, it encapsulates the information into a fixed format message and report the message to its CH during the communication cycle. Also, the message includes the observation results and residual energy of the node. And then, the probability of transferring into active for sensor node and its modules is estimated by two steps: one is that the CH calculates and sends the transferred factors to its members, and the other is the CMs execute the estimation and control the sleep state of their modules based on the received information from their CH.

#### 4.3.1. Calculations of the Transferred Factors by CH

Cluster heads can calculate the transferred factors to their members using information gathered from its members and neighbor CHs. The transferred factors include three parameters, the number of the nodes who sensed a target, the state uncertainty of a target and energy balanced parameter.

(1) The number of the neighbor nodes who sensed a target

For node *N_i_* in a cluster, the number of the *N_i_* neighbors who sensed a target can be obtained from the information received by the CH as follow,

(11) $$n_{s}(N_{i})=\sum_{l=1}^{cm}dr(N_{l},t)+\sum_{f=1}^{ch}dr(Cl_{f},t),N_{l}\in NN_{i}\;$$

where *cm* denotes the number of the CMs in the cluster, *ch* denotes the number of the CHs which send detected information to the current CH, $dr(Cl_{f},t)$ denotes the number of the CMs which are the neighbor nodes of *N_i_* and detect the target in the cluster *Cl_f_*.

(2) The state uncertainty of a target

**Figure 6:**
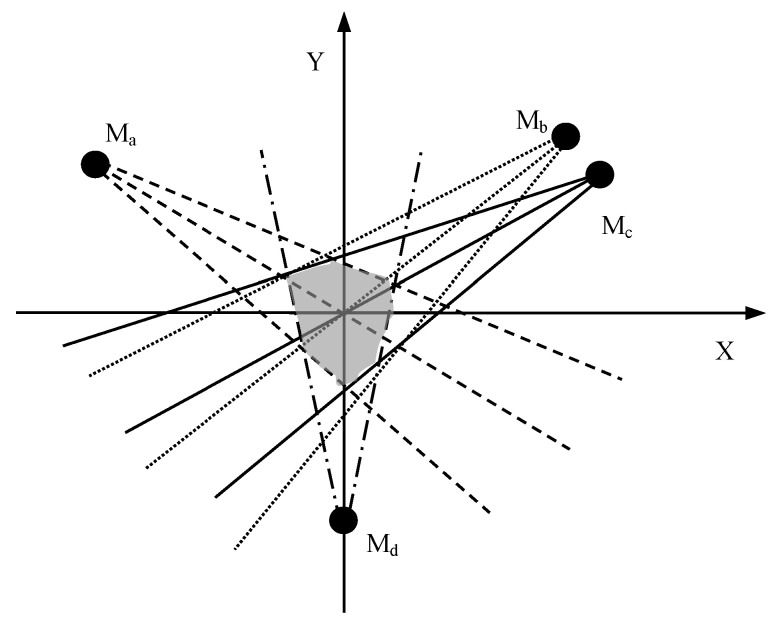
The state uncertainty of the target estimation.

The state uncertainty of a target is estimated by the CH based on the sensing areas of the multiple active sensed modules from different nodes. We represent the position of the node and the sensing range of the sensing module in the coordinate system, as Figure 6 shows. Then, the state uncertainty of the target is regarded as the overlap area of the sight lines of the sensors that can detect the target. For example, $w_{a}SA(M_{a})\cap w_{b}SA(M_{b})$ indicates the weighted intersecting area of the uncertainty area of module *M_a_* and *M_b_*. The smaller the area $w_{a}SA(M_{a})\cap w_{b}SA(M_{b})$ is, the more certainty of the target state estimation by using the sensed information of module *M_a_* and *M_b_* there will be.

In addition, each sensed module *M_j_* of a node has different sensing performance, thus it is set a weight $w_{j}$ according to its detecting accuracy. And then at the time step *t*, the fused state uncertainty result of the *m* sensed modules from multiple nodes is estimated by the cluster head *CH_k_*, and it can expressed as,

(12) $$\begin{array}{ll} f_{su}(CH_{k},t)= & st(M_{1},t)dr(M_{1},t)w_{1}SA(M_{1})\cap st(M_{2},t)dr(M_{2},t)w_{2}SA(M_{2})\cap \ldots \\ & \cap st(M_{m},t)dr(M_{m},t)w_{j}SA(M_{m}) \end{array}$$

where $st(M_{j},t)$ denotes that the sensed module *M_j_* is in sleep or active state when $st(M_{j},t)=0$ or $st(M_{j},t)=1$ respectively, the smaller the fused state uncertainty result is, the more accurate tracking results we can obtain. And $w_{j}$ is the weight value of *M_j_*. It can be obtained as follow,

(13) $$w_{j}=\left\{ \begin{array}{ll} \lambda_{j}da_{j}/\sum_{j=1}^{m}st(M_{j},t)\lambda_{j}da_{j} & \;\mathrm{when}\;M_{j}\;\mathrm{is}\;\mathrm{active}\\ 0 & \;\mathrm{when}\;M_{j}\;\mathrm{is}\;\mathrm{sleep} \end{array}\right.\;$$

where $da_{j}$ indicates the detecting accuracy of *M_j_*, it depends upon the performance and parameters of the sensor.

(3) Energy balanced parameter

To balance the energy consumption and prolong the network lifetime, we have to promote even energy distribution among sensor nodes, thus an energy balanced measurement metric is adopted to decide the energy balanced performance. Let $E_{i}(t)$ be the residual energy of node *N_i_* at current time step *t*, and then in a cluster at time step *t*, the energy balance measurement metric *E_bm_* is calculated by cluster head *CH_k_* using following equation,

(14) $$E_{bm}(CH_{k},t)=\frac{1}{cm}\sum_{i=1}^{cm}{\left(E_{i}(t)-\bar{E}(t)\right)}^{2},\;\bar{E}(t)=\frac{1}{cm}\sum_{i=1}^{cm}E_{i}(t)\;$$

where *cm* denotes the number of CMs in the cluster, $\bar{E}(t)$ is the average residual energy of the cluster at time step *t*. Consequently, the smaller of the value of $E_{bm}(CH_{k},t)$ is, the more the energy consumption of the network nodes is balanced. Since energy consumption is different for different sensed modules, so that we have to choose the sensors to track the target so as to decrease the value of $E_{bm}(CH_{k},t)$ as much as possible. We use $E_{bm-M_{j}}(CH_{k},t+1)$ to represent the energy balance measurement metric at the end of *t* + 1 time step when sensed module *M_j_* is turned on at *t* + 1 time step. Subsequently, in the cluster, the CH estimates the energy balance measurement metric $E_{bm-M_{j}}(CH_{k},t+1)$ for each sensed module *M_j_* based on the power consumption of *M_j_*, where j∈[1,sm] and *sm* is the number of the sensed modules in the cluster. Finally, the CH finds out the energy-balanced parameter $E_{bp}(M_{j})$ for each sensed module as follow,

(15) $$E_{bp}(M_{j})=\frac{\min\{E_{bp-M_{1}}(CH_{k},t+1),E_{bp-M_{2}}(CH_{k},t+1),\ldots E_{bp-M_{sm}}(CH_{k},t+1)\}}{E_{bp-M_{j}}(CH_{k},t+1)}\;$$

where $\min\{E_{bp-M_{1}}(CH_{k},t+1),E_{bp-M_{2}}(CH_{k},t+1),\ldots E_{bp-M_{sm}}(CH_{k},t+1)\}$ denotes the minimum value of $E_{bp-M_{j}}(CH_{k},t+1),j\in [1,sm]$, *sm* is the number of the sensed modules in the cluster.

After the CH finishes the transferred factors calculation, it encapsulates the number of the nodes who sensed a target, the state uncertainty of a target and energy balanced parameter into a fixed format scheduling message and sends the message to its members during the communication cycle.

#### 4.3.2. Sleep State Management for Sensor Node Modules by CMs

At the end of the current time step, CM listens to the wireless communication channel and receives the sleep scheduling information from their CHs to arrange the sleep or active state for all their modules in the next time step. After CMs receive the scheduling information, they can calculate the probabilities of transferring into the active state in the next time step using the gathered information as follows,

(16) $$P_{s-a}(M_{j})=\left\{ \begin{array}{ll} 0 & f_{su}(CH_{k},t)\leq th_{su}\\ E_{bp}(M_{j})\frac{f_{su}(CH_{k},t)-th_{su}}{f_{su}(CH_{k},t)}\alpha_{j} & \;0\text{<}n_{s}(N_{i})<th_{n}\;and\;f_{su}(CH_{k},t)\leq th_{su}\\ 0 & n_{s}(N_{i})=0\;or\;n_{s}(N_{i})\geq th_{n} \end{array}\right.\;$$

where $P_{s-a}(M_{j})$ is the probability of module *M_j_* in active state in the next time step, $th_{su}$ is the threshold of the estimated state uncertainty of a target and it represents the biggest value of the target state uncertainty tolerated by users, and $th_{n}$ is the threshold of the number of nodes which detected the target. $\alpha_{j}$ is a detected accuracy factor and $\alpha_{j}=P(M_{j})\;da_{j}$, and it reflects the detected performance of the sensed module. At the end of the current time step, each CM generates a random number $Ran(M_{j})\in [0,1]$ for its sensed module *M_j_*. If $Ran(M_{j})\leq P_{s-a}(M_{j})$, *M_j_* will be active in the next time step, or else, it will sleep. Corresponding to the above probability of switching to active state, the probability of the sensed module switching to sleep mode in the next time step is $1-P_{s-a}(M_{j})$, and the sleep time is $T_{s}(N_{i})$ calculated in Section 4.1.

From Equation (16), it can be seen that the sensed modules of node *N_i_* can get into sleep when $n_{s}(N_{i})=0$. That is because $n_{s}(N_{i})=0$ means there is no neighbors of node *N_i_* detected a target, that is, no target appears in the deployed area of the neighborhood of *N_i_*, thus node *N_i_* does not need to turn on its sensed modules. Otherwise, when $n_{s}(N_{i})\geq th_{n}$ or $f_{su}(CH_{k},t)\leq th_{su}$, it means there are enough sensor nodes and sensed modules can detect the target, it is not necessary to turn on more sensed modules, thus the probability of switching to active mode for sensed module *M_j_* in the next time step is 0. When some of the neighbors of node *N_i_* detect the target successfully, it denotes the target is actually within the sensing range of the neighborhood of *N_i_*. In order to achieve the required tracking accuracy, node *N_i_* should increase the probability of its sensed modules entering active mode and turn on some of its sensed modules in time. Moreover, the probability of the sensed module entering active mode is also affected by the detected performance, the energy balanced parameter and the target state uncertainty. The better detected performance the sensed module has, the higher probability of entering active mode it has. Accordingly, if turning on the sensed module is enabled to increase the energy balanced parameter, the sensed module has more probability entering active mode, and if the target state has less certainty, the sensed module has more probability to be turned on.

In addition, if a node is in the set of *TC*(*t*) and detects a target in the current time step, it continues to maintain active mode and its communication and MCU module are also active in the next time step. If the sensed modules of a node are all in sleep mode and its communication and MCU modules are also turned off in the next time step, and the sleep time is $T_{s}(N_{i})$. Then, there is a timer inside the node to accomplish the sleep task. In addition, after the sleep time is expired in a node, the communication and MCU modules switch into active mode to receive the information from its CH and arrange the sleep scheduling for all its modules in the next time step. The sleep scheduling procedures of EBMS algorithm is shown in Algorithm 1. Using this way not only can balance the energy consumption of each node, but also can obtain the required tracking accuracy with less energy consumption by selecting some of sensed modules to execute the tracking tasks.
**Algorithm 1.** The sleep scheduling procedure of node *N_i_*.1. **input** the threshold *th_su_*, *th_n_*2. **receive** sleep scheduling information from its CH3. **extract**
*n_s_*(*N_i_*), *f_su_*(*CH_k_*, *t*), and *E_bp_*(*M_j_*)4. **if** (*dr*(*N_i_*,*t*) = 1)5.  **for each** module of *N_i_*6.   **get into** active (*N_i_*, *t* + 1)7. **else if** ($f_{su}(CH_{k},t)\leq th_{su}$ or $n_{s}(N_{i})=0$ or $n_{s}(N_{i})\geq th_{n}$)8.  **for each** module of *N_i_*9.   **get into** sleep (*N_i_*, *t* + 1)10.    **while** sleep time $T_{s}(N_{i})$ is expired11.     **get into** active state st_2_12.     **wake up** (MCU and communication modules)13. **else if** ($0\text{<}n_{s}(N_{i})<th_{n}\;and\;f_{su}(CH_{k},t)\leq th_{su}$)14.  **for each** sensed module *M_j_*15.   **calculate**16.    $P_{s-a}(M_{j})=E_{bp}(M_{j})(f_{su}(CH_{k},t)-th_{su})\alpha_{j}/f_{su}(CH_{k},t)$17.   **generate**
$Ran(M_{j})\in [0,1]$18.   **if** ($Ran(M_{j})\leq P_{s-a}(M_{j})$)19.    **get into** active (*M_j_*, *t* + 1) 20.   **else**21.    **get into** sleep (*M_j_*, *t* + 1)22. **end for**

If the proposed approach is used in other sensing application case, we need to know which sensors are used in the application and how many sensors a node has. In addition, the power consumption of each sensor, the sensing range of each sensor, and the sensing accuracy of each sensor should be aware. It is convenient to use the aforementioned information to make the sleep scheduling for sensor nodes.

If the application requires only one sensor per node, the benefits of this scheme are to select an appropriate sensor in each node according to the residual energy of the node and the power consumption of each sensor in the node. For example, selecting the video sensor in Node *N_x_* and the ranging sensor in Node *N_y_* to sense a target. That is, in this scheme, the nodes around the target and their sensors are coordinated to meet the requirements of tracking and balance the energy consumption of each node.

In the EBMS algorithm, the state distribution of nodes and their sensed modules in time step *t* is only related to the state of them in time step *t* − 1, but it has no relation to the state distribution before the time step *t* − 1. Therefore, it is a typical Markov process [23], which is a discrete random distribution process, and the discrete random sequence can be described by Markov chains. In this paper, the state of each node can be described by a Markov chain, which represents a series of states, *K*_1_, *K*_2_, …, *K_t_*, and these states are contained in the state space set *V*, which consists of two subsets *S* (sleep mode) and *A* (active mode). Subset *S* is composed of *d* states, which are *s*_1_, *s*_2_, …, *s_d_*, respectively. And each state represents the different sleep mode in which sensor nodes have the different residual sleep time. Subset *A* includes three small subsets *Al*, *As* and *Au*. *Al* includes *e* states, which are *Al*_1_, *Al*_2_, …, *Al_e_*, and each state represents the different listening mode in which the nodes have the different number of neighbors who detect a target. *As* denotes the node is in active mode and successfully detects a target in time step *t*, and the node keeps in active mode until the target is out of its maximum sensed range. Therefore, *As* consists of *f =* 2*max_R_s_*/*v_max_* status values, which are *As*_1_, *As*_2_, *…*, *As_f_*. *Au* represents the node is in active mode and detects no target in the time step *t*, and then, the running state Markov chain of the node is shown as followings,

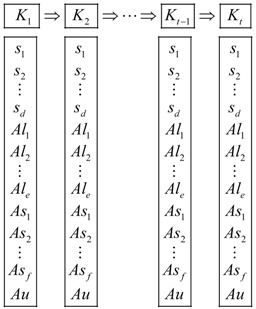
(17)

Consequently, the sensor node, which has maximum sleep time *t_d_* and neighbor nodes *n_e_* and its active periods are the time step from 0 to *f*, has a set with dimension *D = t_d_* + *n_e_* + *f* + 1. For a sleeping node, the probability of state transferring within the subset *S* is,

(18) $$P(s_{t\_x}|s_{(t-1)\_y})=\left\{ \begin{array}{ll} 1, & x=y-1\;and\;1\leq y\leq t_{d}\\ 0, & \;\mathrm{others} \end{array}\right.\;$$

When the sleep time of a node expires and the node transfers into active mode to listen and receive information from its CH, and the probability of the node and its sensed modules switching to sleep or active mode in the next time step depends on the probabilistic value $P_{s-a}(M_{j})$. If a WSN has *n* nodes and every node has *m* sensed modules, and the probability of a target simultaneously sensed by *ms* sensed modules at least in the time step *t* is,

(19) $$P(m\times n,ms,t)=1-\sum_{\mu =0}^{ms}{\left(C_{m\times n-\mu }^{m\times n}(1-P(As_{t}))\right)}^{m\times n-\mu }{\left(P(As_{t})\right)}^{\mu })\;$$

In addition, we assume a target can be successfully tracked, if there are more than *th_ms_* sensed modules detecting it. Thus, in this paper, the tracking success rate (TSR) refers to the ratio of the time when the target is under the detection of *th_ms_* sensed modules and the total time that the target is in the deployed area.

(20) $$TSR=\frac{1}{t_{h}}\sum_{t=0}^{t_{h}}P(m\times n,th_{ms},t)\;$$

### 4.4. The Discussion on Multi-Hop Collaboration of CHs

**Figure 7:**
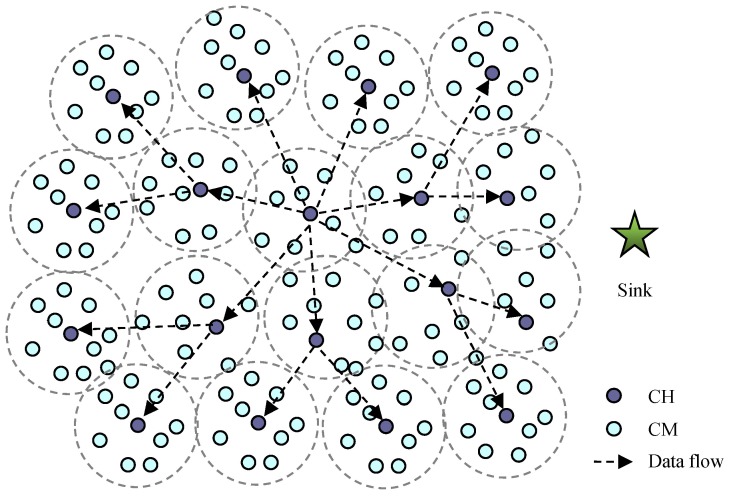
Illustration a clustering structured sensor network.

In above Section 4.1, when CHs calculate the sleep time and make the scheduling management decisions for their members, they consider the detected information from their immediate neighbor CHs. Here, we can also increase the coordination among the CHs located within a multi-hop communicated range, that is, a CH can use the information of the target from its neighbor CHs. Then, the CH receives the detected information from the CHs located at *h*-hop away from itself. The CH sends the detected information of its cluster to its neighbor CHs, which relay the information to the next hop CHs until the information arrives at the *h*-hop CHs, as Figure 7 shows. The detected information includes the location of the sender and the distance between the sender and the target. Consequently, the CH can arrange the sleep time of its members according to the gathered information. If the information denotes a target is detected, the sleep scheduling of the CMs remain unchanged, otherwise, if the information denotes no target is detected, the CH can estimate the sleep time for its members by the following equation.

(21) $$T_{s\_h}(N_{i})=T_{s}(N_{i})+(h-1)\frac{\min\_d(CH_{u},CH_{v})}{v_{\max}}\;$$

where $\min\_d(CH_{u},CH_{v})$ is the minimum distance between the current *CH_v_* and its neighbor *CH_u_*, *h* is the maximum number of the detected information transmitted hops. From Equation (21), it is seen that the CMs have more sleep time when no targets are found around the cluster. This is because the CH knows no target is in the area with an *h*-hop radius, and its members can sleep longer time to save energy without missing a target. However, it takes energy to transmit the detected information to multi hops. Thus, we should find out a balance between the energy consumption of the detected information transmitting and the energy saving of the CMs sleeping. We assume the energy consumption of sending and receiving the detected information are *E_se_* and *E_re_*, respectively. If CHs consider the detected information only from their immediate neighbor CHs, the energy consumption for data transmitting is,

(22) $$E_{c}(1)=E_{se}+n_{1}E_{re}\;$$

where *n*_1_ is the number of the immediate neighbor CHs of the current cluster, if the cooperation among the CHs is extended to *h*-hop neighbors, the CHs send data to the *h*-hop neighbor CHs and also receive data from them, then the energy consumption for data transmitting is obtained as followings,

(23) $$E_{c}(h)=E_{c}(1)+\sum_{q=2}^{h}(n_{q-1})E_{se}+\sum_{q=2}^{h}n_{q}E_{re}\;$$

where *n_q_* is the number of the neighbor CHs in the *q-*th hop transmission, the CHs need to receive the detected data. On the contrary, if *h =* 1, the energy saving by CMs sleeping is,

(24) $$E_{s}(1)=[E_{a-s}+E_{s-a}+(P_{a}(N_{i})-P_{s}(N_{i}))T_{s}(N_{i})]cm_{a}\;$$

where $P_{a}(N_{i})$ and $P_{s}(N_{i})$ are the power consumption of node *N_i_* in active and sleep state, and *cm_a_* is the average number of CMs in each cluster.

Similar to *h =* 1, if the cooperation among the CHs is extended to *h*-hop neighbors, the energy saving by CMs sleeping can be calculated as,

(25) $$E_{s}(h)=[E_{a-s}+E_{s-a}+(P_{a}(N_{i})-P_{s}(N_{i}))T_{s\_h}(N_{i})]cm_{a}\;$$

Therefore, the energy saving by *h*-hop cooperation is obtained as followings,

(26) $$E_{s}(h)-E_{c}(h)=E_{tr}+(P_{a}(N_{i})-P_{s}(N_{i}))T_{s\_h}(N_{i})cm_{a}-[E_{c}(1)+\sum_{q=2}^{h}(n_{q-1})E_{se}+\sum_{q=2}^{h}n_{q}E_{re}]\;$$

where *E_tr_* is a constant which denotes the energy costs of the nodes state transition,

(27) $$E_{tr}=(E_{a-s}+E_{s-a})cm_{a}\;$$

Since sensor nodes are deployed randomly and uniformly, we assume $n_{q}=qn_{1}$, and then combining Equations (21) and (26), $E_{s}(h)-E_{c}(h)$ can be calculated as followings,

(28) $$\begin{array}{l} E_{s}(h)-E_{c}(h)\\ =(P_{a}(N_{i})-P_{s}(N_{i}))(h-1)cm_{a}\frac{\min\_d(CH_{u},CH_{j})}{v_{\max}}-[\sum_{q=2}^{h}((q-1)n_{1})E_{se}+\sum_{q=2}^{h}qn_{1}E_{re}]+C\\ =(P_{a}(N_{i})-P_{s}(N_{i}))(h-1)cm_{a}\frac{\min\_d(CH_{u},CH_{j})}{v_{\max}}-n_{1}(h-1)[\frac{1}{2}(h+2)(E_{se}+E_{re})-E_{se}]+C \end{array}$$

where $C=E_{tr}+(P_{a}(N_{i})-P_{s}(N_{i}))T_{s}(N_{i})cm_{a}-E_{c}(1)$ is a constant, it is not related with *h*.

To optimize the saved energy, $E_{s}(h)-E_{c}(h)$ should be maximum. Since $P_{a}(N_{i})-P_{s}(N_{i})>0$ and $cm_{a}>0$, $E_{s}(h)-E_{c}(h)$ is differentiated as followings to find the maximum value,

(29) $$\frac{\partial (E_{s}(h)-E_{c}(h))}{\partial h}=(P_{a}(N_{i})-P_{s}(N_{i}))\frac{\min\_d(CH_{u},CH_{j})}{v_{\max}}cm_{a}-n_{1}[(h+\frac{1}{2})(E_{se}+E_{re})-E_{se}]\;$$

Thus, if $\partial (E_{s}(h)-E_{c}(h))/\partial h=0$, we have maximum $E_{s}(h)-E_{c}(h)$ and *h* can be obtained as followings,

(30) $$h=\frac{1}{(E_{se}+E_{re})n_{1}}[\frac{(P_{a}(N_{i})-P_{s}(N_{i}))\min\_d(CH_{u},CH_{j})cm_{a}}{v_{\max}}+n_{1}E_{se}]-\frac{1}{2}\;$$

Therefore, an appropriate *h* value can be chosen to optimize the saved energy by the cooperation among the CHs based on the network parameters. Also, we should consider the costs of reforming cluster structures due to the excessive transmitting energy consumption of CHs in realization. Cluster heads inform their CMs about the updated sleep time if there are changes on the sleep time. And the CMs receive a new sleep time to adjust their sleep mode adaptively.

## 5. Experimental Results and Analysis

In this section, we evaluate and analyze the performances of the proposed scheduling algorithms in different network conditions. We conduct the simulations by Castalia [24] based on OMNet++4.1 [25], which provides realistic and accurate wireless channel models, radio models (CC2420 and CC1000), and MAC models (IEEE 802.15.4) so that the simulation results would become meaningful [26]. We compare the proposed algorithm EBMS with the state-of-the-art approaches SSA-DC (Simple node Scheduling Approach in view of Data Correlation) [4], OBSP (Optimized Back-off Sleep Protocol) [11], and DSDA (Distributed Saturation Degree-based Algorithm) [13] by running them in the same networks with same parameters in the simulations.

### 5.1. Experiment Environment

In our simulation, the WSN included 300 sensor nodes randomly deployed in a 200 m × 200 m area, and every node had 1 J (Joules) initial energy and 3 sensed modules, and the observation error of every sensor followed a Gaussian distribution. The other parameters and their values used in our simulations are summarized in Table 3.

The dynamical state model of a target was defined by a 4-dimensional state vector $X_{k}={\left[x_{k},v_{kx},y_{k},v_{ky}\right]}^{T}$ where $\left(x_{k},y_{k}\right)$ was the location of the target at time instant *k* and $v_{kx},v_{ky}$ were the velocities of the target in the directions of *x*- and *y-*axis, respectively. The model of the target motion was assumed to be $X_{k+1}=QX_{k}+P_{k}$ where *Q* was the state transition model and *P_k_* was the process noise which was assumed to be Gaussian with mean zero. The state transition model of a target is given as follows,

(31) $$X_{k+1}=\left[\begin{array}{cccc} 1 & T & 0 & 0\\ 0 & 1 & 0 & 0\\ 0 & 0 & 1 & T\\ 0 & 0 & 0 & 1 \end{array}\right]X_{k}+\left[\begin{array}{c} T^{2}/2\\ T\\ T^{2}/2\\ T \end{array}\right]v_{k}\;$$

where *T* was the sampling time interval, *T =* 1 s. $v_{k}$ denotes the noise of the target state transition, $v_{k}\sim N(0,\sigma )$, $\sigma =2^{{}^{\circ}}$. The target changed its position randomly based on a maximum acceleration *a_max_ =* 2 m/s^2^ and a maximum speed *v_max_ =* 10 m/s. The initial state of the target was assumed to be $X_{0}={\left[0,3,0,3\right]}^{T}$.

### 5.2. Simulation Results

**Figure 8:**
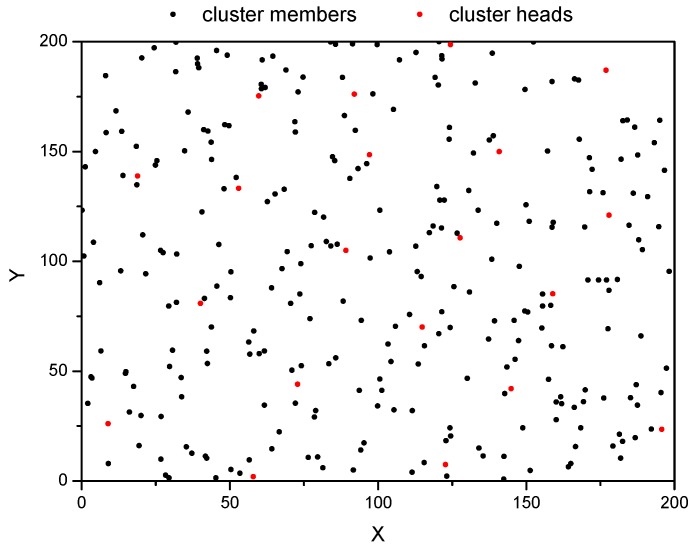
The deployment of the sensor nodes and cluster heads.

In this section, we illustrate the performance of the proposed sensor scheduling algorithm by numerical examples. Figure 8 shows a scene of the two-dimensional sensing area where 300 multisensory sensor nodes were randomly distributed. Also, the clustering network structure was formed, and the nodes were divided into CHs and CMs.

**Figure 9:**
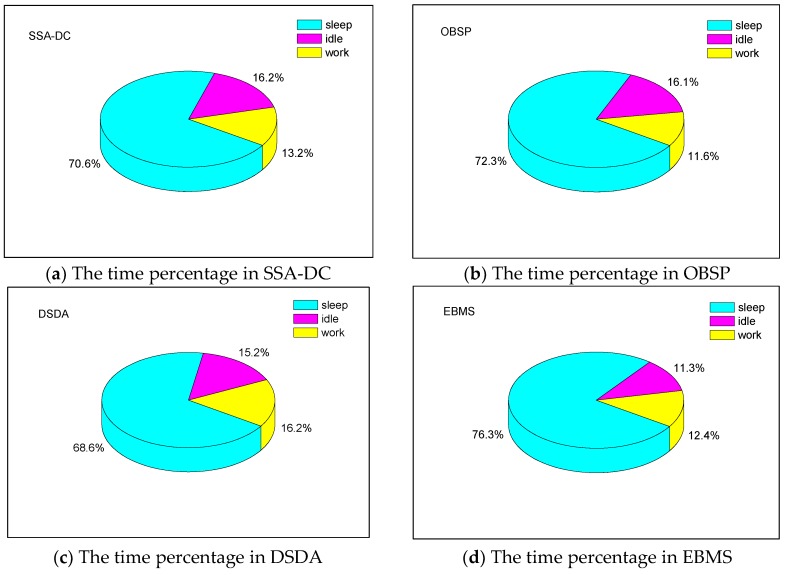
The time percentage of sleep and activity of nodes.

The average time percentages of sleep and activity of each module in sensor nodes under the different scheduling algorithms are illustrated in Figure 9. The time percentage of the idle, sleep, and work is the ratio of the period when a module was in idle, sleep, and work status to the total simulation time, respectively. The idle and work statuses denote the module was active. Although the module in the idle status was active, it had not sensed or communicated tasks. From Figure 9, it can be seen that EBMS effectively reduced the idle time and increased the sleep time of modules in the multisensory nodes compared with the other three algorithms. As shown, using EBMS the average sleep time percentage of the modules was 76.3%, while SSA-DC, OBSP and DSDA allowed the modules to sleep 70.6%, 72.3%, and 68.6%, respectively. It was because an adaptive sleep time allocated scheme based on the distance between the CM and the cluster border was adopted in EBMS to maximize the sleep time of CMs. Moreover, EBMS can adaptively arrange the scheduling for communicated modules as well as the sensed modules of nodes so that the energy intensive sensed modules can sleep more to save energy. Nevertheless, the sensed module scheduling was not involved in the SSA-DC, OBSP, and DSDA approaches, and they have lower sleep time percentages. In OBSP, since each node randomly and independently decides whether to become active or sleep and it does not consider the information from its neighboring area, the modules have a lot of idle time. The distributed saturation degree-based algorithm DSDA can reduce the idle time percentage by the cooperation among the adjacent nodes. However, it needs massive sensing information transmissions among the nodes so that the working time percentage of the modules increased. In addition, the working time percentage in SSA-DC is higher than that in OBSP because the sensed data correlation is considered in the node scheduling so as to raise the number of data processing and transmitting. Figure 9 also explains why EBMS is the most energy efficient compared to the other three algorithms.

**Figure 10:**
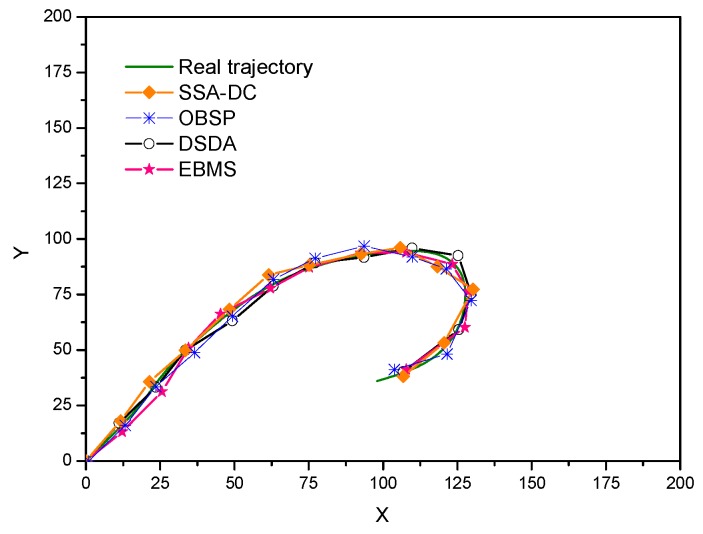
Target trajectories estimation under different scheduling algorithms.

**Figure 11:**
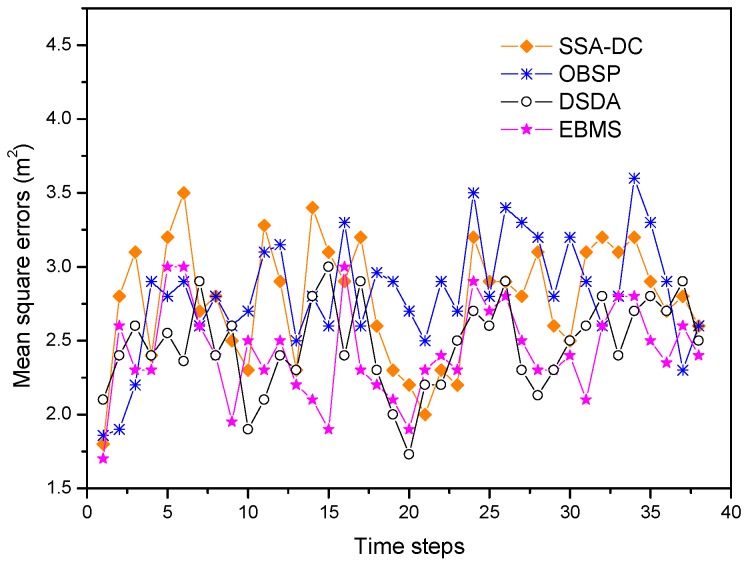
Mean square positioning errors under different methods.

Figure 10 draws the real and estimated position of the moving target for the different scheduling methods. Moreover, the mean square error between the real trajectory and estimated position of the target at each time step in these scheduling methods is shown Figure 11.

In OBSP, since the number of target tracking nodes is not enough when the direction of the target changes especially and the observation relation of the nodes are not considered, the accuracy of OBSP is worse than the other three approaches. Due to considering the sensed data correlation among the adjacent nodes, the tracking error of SSA-DC decreases at the later time steps and the accuracy will be better than that in OBSP. In contrast, the tracking performances of EBMS and DSDA are superior to those of SSA-DC and OBSP approaches. The distributed saturation degree-based algorithm (DSDA) always schedules enough active nodes and there is efficient cooperation among nodes, so the tracking error is the smallest and the target tracking accuracy is the best. We can see that the proposed EBMS shows similar tracking performance as that in the DSDA approach. This is because EBMS ensures there are enough sensed modules involved in target tracking by using the target state uncertainty factor. Moreover, CHs can use the results of the target detection from its neighbor CHs by the cooperation among the CHs, thus EBMS can obtain the good tracking performance.

**Figure 12:**
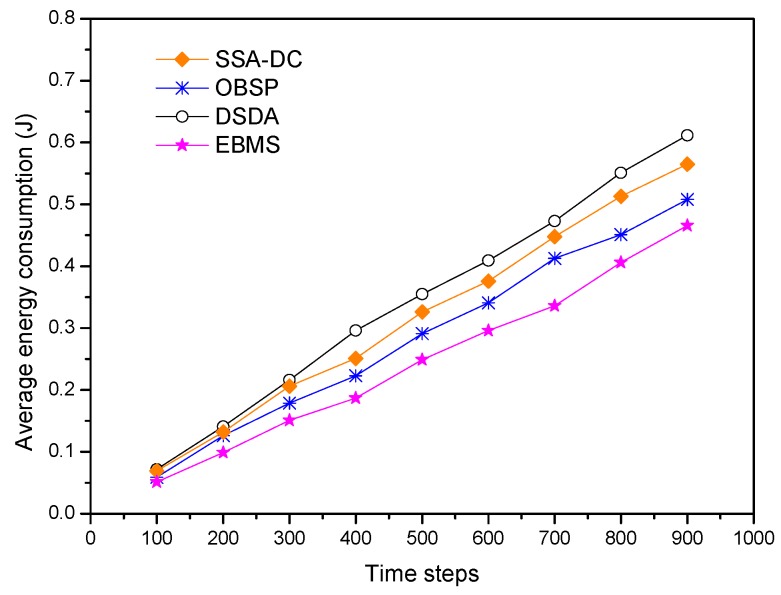
Average energy consumption under different scheduling algorithms.

Figure 12 shows the average energy consumption changed with simulation time steps in the different scheduling algorithms. It can be seen that EBMS consumes the least energy and has the best energy efficiency compared to the other three approaches. The reasons for this are summarized as follows: First, CHs adaptively arrange the sleep time of its members according to the location of the members to maximize the sleep time of each CM. Second, both communication and sensed modules of the nodes can be efficiently scheduled in EBMS. Moreover, the optimal collaboration is established among clusters in a coordinated way. Therefore, the detected information is just transmitted among the CHs instead of all the nodes so as to maximize the energy conservation. In contrast, the other three scheduling algorithms do not consider the scheduling management of the sensed modules of the multisensory nodes so that they consume more energy than EBMS. The DSDA and SSA-DC approaches have more energy consumption than OBSP because they consider the data correlation and cooperation among the adjacent sensor nodes so that more communication cost among neighbor nodes are involved. We can see that EBMS achieves about 17.5% and 23.9% energy saving compared with SSA-DC and DSDA, respectively.

**Figure 13:**
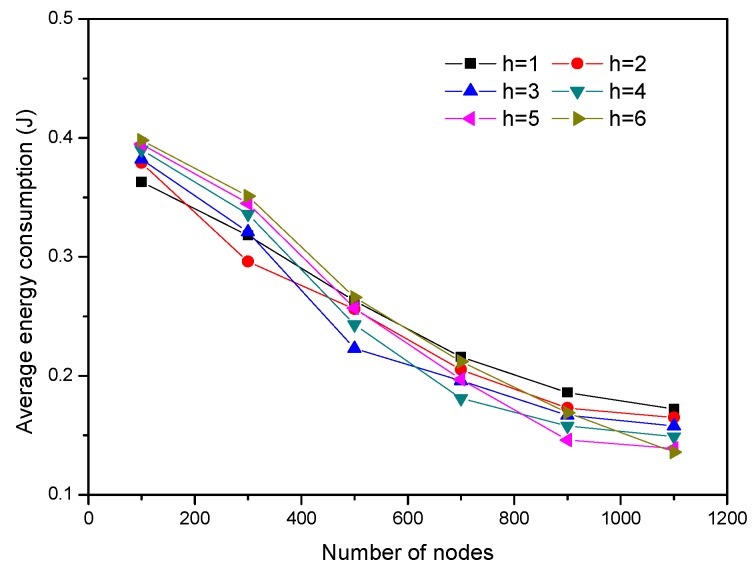
Average energy consumption with different node densities.

Here, we investigated the impact of coordinated hops among the CHs on the energy efficiency of EBMS, by changing the node number from 100 to 1100. A comparison of the average energy consumption for different coordinated hops *h* is illustrated in Figure 13. When CHs calculate the sleep time and make the scheduling management decisions for their members, they can use the detected information from their *h*-hop neighbor CHs. That is, a CH receives the detected information from the CHs located at *h*-hop away from itself. Simultaneously, the CH sends the detected information to its *h*-hop neighbor CHs. As Figure 13 shows, if the number of nodes is 100, the average energy consumption is the least when *h =* 1. If the value of *h* increases, the cost of communication among the CHs increases. As a result, the amount of average energy consumption rises. However, if the number of nodes is 300, the average energy consumption is the least when *h* = 2. This is because the average number of nodes in one cluster is small when node density is small. Then, the energy saved by the sleep of CMs is smaller than that consumed by the communication of CHs. By comparison, the average number of nodes in one cluster is large when the nodes are high density, which results the energy saved by the sleep of CMs is bigger than that consumed by the communication among the CHs. For example, when the number of nodes is 900, the average energy consumption is the least if *h =* 5. Therefore, we can choose the appropriate *h* to obtain the optimal energy conservation.

**Figure 14:**
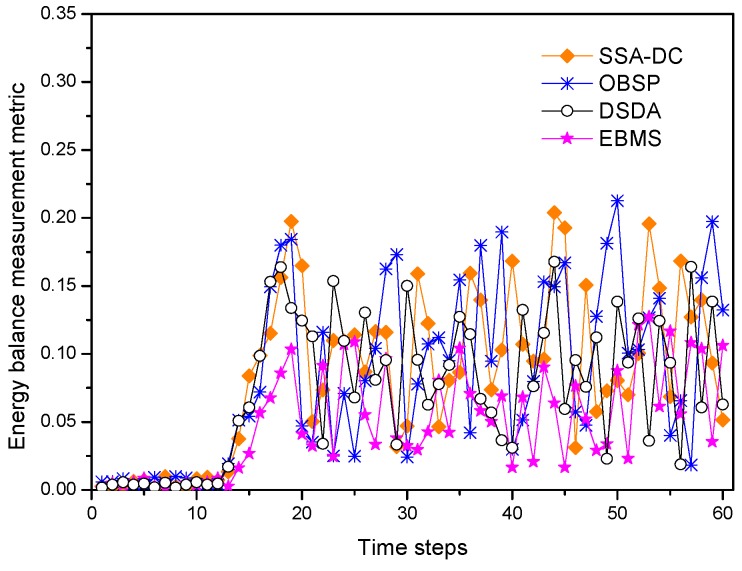
Energy balance measurement metric under different scheduling algorithms.

Figure 14 shows the energy balance measurement metric of the network changed with simulation time steps under the different scheduling algorithms. The energy balance measurement metric illustrates whether the energy consumption of nodes in the whole network is balanced or not, and it is calculated as follows,

(32) $$E_{bm}(t)=\frac{1}{n}\sum_{i=1}^{n}{\left(E_{i}(t)-\bar{E}(t)\right)}^{2},\;\bar{E}(t)=\frac{1}{n}\sum_{i=1}^{n}E_{i}(t)\;$$

where *n* denotes the number of sensor nodes in the network, $\bar{E}(t)$ is the average residual energy of the nodes at time step *t*. $E_{i}(t)$ is the residual energy of node *N_i_* at current time step *t*. From Figure 14, we can see that the energy balance metric are near to zero at the initial time step in all the algorithms. This is because the nodes have the same initial energy and little energy of the node is consumed in the initial phase so that the network has a balance energy consumption. However, as time goes on, the nodes near to the target are active frequently so that they consume a lot of energy, and then the energy consumption is becoming more and more unbalanced. Compared with SSA-DC and OBSP, DSDA distributes energy consumption among nodes in the sensing range of the target to avoid scheduling frequently lower-energy nodes, as such, DSDA obtains better energy-balanced performance than SSA-DC and OBSP. In contrast, the nodes located in cluster center to execute the energy-intensive sensing or data transmitted task in EBMS since these nodes can sleep more. Furthermore, EBMS can adaptively arrange the scheduling for the sensed modules of nodes so that the energy intensive sensed modules can sleep more. Therefore, EBMS achieves the best energy-balanced performance compared with the other algorithms.

**Figure 15:**
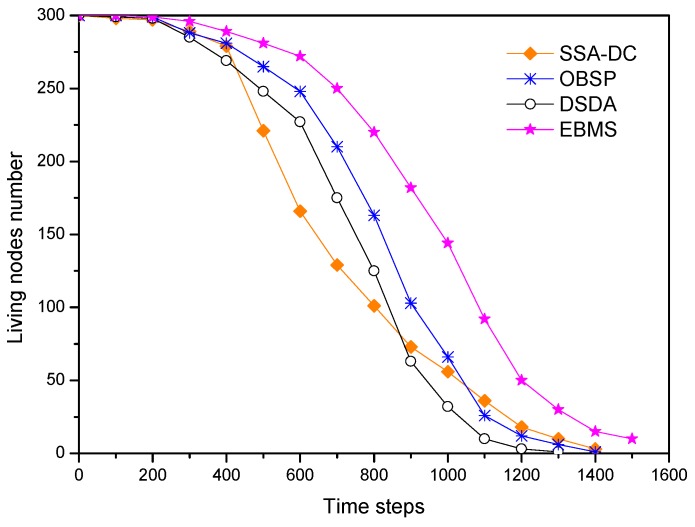
Lifetime under the different scheduling algorithms.

Figure 15 shows the number of the living nodes versus the simulation time steps under the different scheduling algorithms. Due to less energy cost, the number of living nodes in SSA-DC is more than that in DSDA in the initial period. However, the energy consumption in SSA-DC is unbalanced as time goes on so that the number of the energy-depleted nodes is growing rapidly, and then the living nodes in SSA-DC are less than that in DSDA in the following time steps. Compared with SSA-DC and DSDA, OBSP has lower average energy consumption of each node and the total energy consumption, thus the nodes can obtain a longer lifespan in OBSP. As shown, EBMS can significantly prolong the lifetime of the nodes compared with the other algorithms. For example, EBMS achieves the number of living nodes increasing by 63% and 35% compared with DSDA and OBSP in the 800th time steps. There are three main reasons for the node lifetime extension. First, EBMS adopts adaptive sleep scheduling for CMs and the CMs in the interior of a cluster have long sleep time. Second, the sensed modules of the multisensory nodes are efficiently arranged by scheduling the optimal sleep time. Third, the energy consumption of the nodes is balanced. Therefore, the proposed EBMS can effectively prolong the network lifetime.

The proposed approach EBMS cannot execute target-tracking tasks with a reliable tracking accuracy when the number of living nodes drops to 10%. At that time, the living nodes can be active to sense and communicate. However, the density of the living nodes reduces and the distance between the remaining nodes increases, the remaining nodes may send messages unsuccessfully or with high-energy consumption. Figure 15 shows only the trend of the number of the exhausted nodes when there are very few living nodes, and it indicates that the fewer nodes remain, the faster the death rate of the nodes becomes, because the remaining nodes have longer communication distances.

**Figure 16:**
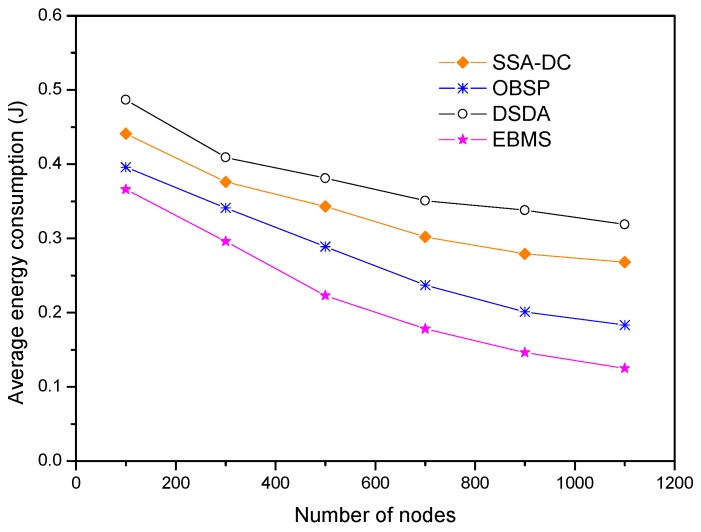
Average energy consumption under the different node densities.

The number of nodes in the network varies from 100 to 1100 with an increment of 200. The simulation results are demonstrated in Figure 16. It shows that the average energy consumption of sensor nodes descends when the node density increases. This trend is obvious in the proposed EBMS. It is because a given set of selected nodes and modules can achieve the demanded target tracking performance, and more nodes and their modules can be in sleep mode when the network is densely deployed. In this way, the size of selected tracking nodes and sensed modules keeps stable compared with the increment of nodes in the network, and the newly added nodes can sleep more. In contrast, the average energy consumption of nodes in SSA-DC and DSDA decreases slightly when the node density increases. This is because more communication costs among the neighboring nodes are involved to transmit the detected information, and the correlation of the detected results is complicated when the nodes have a higher density. The increasing node number causes the slight decrease in average energy consumption.

Because only a certain number of nodes needs to be in active state to track the moving target, some extra nodes are turned off to save energy when the node density increases. Thus, the number of nodes in sleep state increases relatively so that the average energy consumption decreases when the node density increases. That is, if we want to extend the lifetime of the network, it can be achieved by increasing the density of nodes.

**Figure 17:**
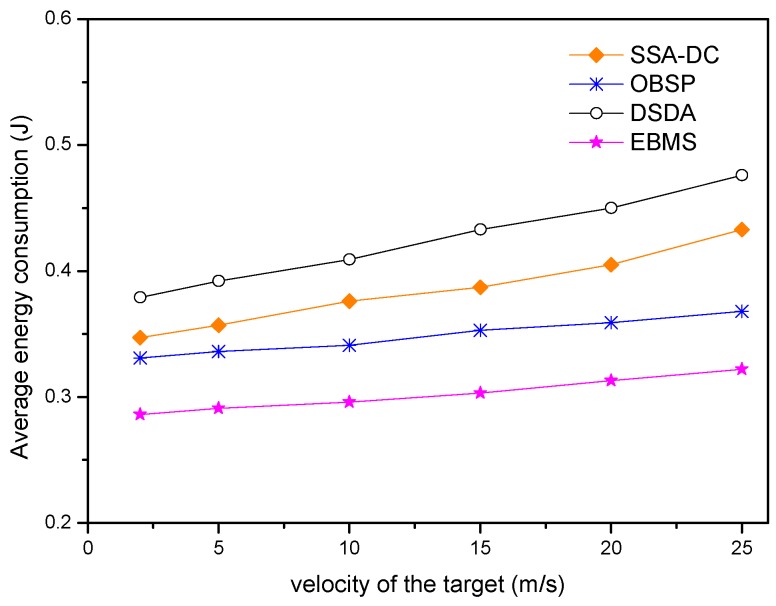
Average energy consumption under the different target velocities.

Figure 17 shows the energy cost changed with target velocity under the different scheduling algorithms. The value of target velocity varies from 2 m/s to 25 m/s. The results show that the average energy consumptions of EBMS and OBSP ascend slightly when the target velocity increases. This is because the nodes have the same sleep scheduling arrangement in the different target velocities. The slight increase in average energy consumption is caused by data transmissions. However, due to the frequent position changing of the target, more nodes are woken up in DSDA to detect so that it has a significant rise in average energy consumption with the target velocity increasing. The average energy consumption in SSA-DC also rises because it has to consider the correlation of the detected information from more nodes if the target has a higher velocity. As shown in Figure 17, the proposed EBMS always has a high-energy efficiency with a different target velocity. When target velocity increases, sensor nodes and sensed modules can adjust their sleep scheduling adaptively so that the average energy consumption is smaller than that of the other scheduling algorithms. For example, in the case that *v* = 10 m/s, the average energy consumption in EBMS is 0.29 J, and they are 0.34 J, 0.37 J, and 0.41 J by OBSP, SSA-DC, and DSDA respectively.

**Figure 18:**
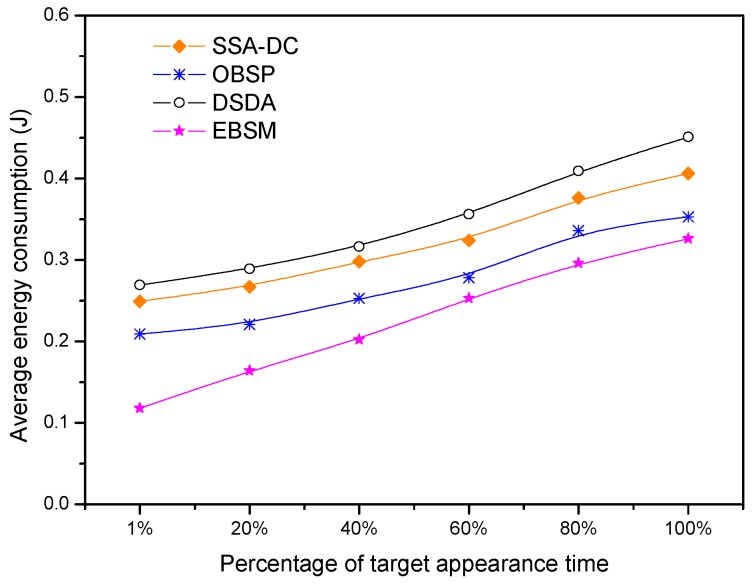
Average energy consumption under the different percentages of the target appearance time.

Since the target is not always present in the target tracking application, we further investigated the relationship between the percentage of the time when the target appears and the average energy consumption under the different scheduling algorithms. The percentage of the target appearance time is varied from 1% to 100%, and the results are shown in Figure 18. We can see that the average energy consumption increases with the percentage of the target appearance time in all the scheduling algorithms. This is because more nodes and their modules are awake and more detected information needs to be transmitted if the target frequently appears. Nevertheless, the average energy consumption of EBMS is the smallest under the different percentages of the target appearance time compared with the other scheduling algorithms. Especially, EBMS consumes significantly less energy if the target appears at a low frequency. This is because EBMS can adaptively adjust the sleep scheduling of the nodes and sensed modules according to the appearance of the target so that more sensor nodes and sensed modules can go to long-term sleep. This feature is very suitable for target tracking applications, because there is often a long time interval between the occurrences of two targets in target tracking applications. Therefore, EBMS can significantly extend the lifetime of the target-tracking network.

## 6. Conclusions

This paper proposed a novel distributed and energy-balanced multisensory scheduling strategy for target tracking WSNs. The scheduling of both nodes and sensed modules were considered in our proposed EBMS to find an optimal nodes cooperation to conserve energy consumption without degrading tracking performance. This was realized by assigning each node and its sensed modules an optimal sleep time according to its position and the target detected information. Meanwhile, EBMS can also be used to balance the energy consumption of sensor nodes by adopting an energy balanced measurement metric in the procedure of multisensory node scheduling. Our experiments illustrated EBMS reduced the average energy consumption as well as prolonged the network lifetime. Also, the experiments proved that EBMS outperformed the state-of-the-art approaches.

## Figures and Tables

**Table 1:** Different combinations of each module state.

States	Sensing	Processing	Memory	Radio
st_0_	Active	Active	Active	Rx/Tx
st_1_	Active	Idle	Sleep	Rx
st_2_	Active	Sleep	Sleep	Sleep
st_3_	Sleep	Sleep	Sleep	Sleep

**Table 2:** Sleep states combinations for the sensors.

Sensing	Sensed Module M_1_	Sensed Module M_2_	Sensed Module M_m_
Active	Active	Active	Active
	Active	Active	Sleep
	Active	Sleep	Sleep
Sleep	Sleep	Sleep	Sleep

**Table 3:** Simulation parameters.

Parameters	Values
Data reporting frequency	1 s
Data packet size	128 bytes
Control message size	8 bytes
*th_su_*	2 m^2^
*th_n_*	10
Coordinate of sink	(200, 200)
*E_elec_*	50 nJ/b
ε_amp_	100 pJ/(b·m^2^)
Active time slot	0.2 s
*P_sens_* of *M*_1_	0.1 mW
*e_a-s_*/*e_s-a_* of *M*_1_	8 × 10^−8^ J
Sensing range *R_sens_* of *M*_1_	15 m
Sight angle of *M*_1_	100°
λ_1_ of *M*_1_	98.60%
*da*_1_ of *M*_1_	97.60%
*P_sens_* of *M*_2_	3 mW
*e_a-s_*/*e_s-a_* of *M*_2_	1.2 × 10^−7^ J
Sensing range *R_sens_* of *M*_2_	5 m
Sight angle of *M*_2_	30°
λ_1_ of *M*_2_	99.30%
*da*_1_ of *M*_2_	98.10%
*P_sens_* of *M*_3_	20 mW
*e_a-s_*/*e_s-a_* of *M*_3_	3.6 × 10^−6^ J
Sensing range *R_sens_* of *M*_3_	8 m
Sight angle of *M*_3_	25°
λ_1_ of *M*_3_	99.60%
*da*_1_ of *M*_3_	98.60%
